# The interleukin-1 cluster, dyslipidaemia and risk of myocardial infarction

**DOI:** 10.1186/1741-7015-8-6

**Published:** 2010-01-13

**Authors:** Bernard Keavney

**Affiliations:** 1 BHF Professor of Cardiology, Institute of Human Genetics, Newcastle University, Newcastle, UK

## Abstract

Coronary heart disease (CHD) is among the most serious worldwide health problems. Recent genetic studies have robustly identified a number of polymorphic loci throughout the genome that are associated with disease risk but it is certain that more remain to be discovered. It is well established that inflammation plays a key role in the pathophysiology of CHD. Determining whether or not polymorphisms in genes involved in the inflammatory cascade affect the risk of CHD is of considerable interest with respect to understanding the direction of the causal arrow between increased expression of inflammatory genes and CHD. Establishing an association between the variation in inflammatory genes and CHD would provide conceptual support for the use of appropriately specific anti-inflammatory agents in CHD prevention and, potentially, suggest new therapeutic targets. This month in *BMC Medicine*, Benjamin Brown and colleagues adopt a family-based case-control association study design to address this question. In a large number of CHD cases and healthy sibling controls genotyped for 51 mainly coding single nucleotide polymorphisms (SNPs), they find evidence for the association of a common haplotype at the Interleukin-1 (IL-1) cluster with CHD which appears to be stronger in younger cases without hypercholesterolaemia. They also find suggestive evidence for an association between this same haplotype and hypercholesterolaemia. If replicated in other cohorts, these results could be of substantial importance in advancing the understanding of the way in which inflammatory genes affect coronary heart disease risk.

See the associated research paper by Brown et al: http://www.biomedcentral.com/1741-7015/8/5

## Commentary

This month in *BMC Medicine*, Benjamin Brown and colleagues present a large genetic study which specifically addresses the effect of polymorphisms in genes involved in the inflammatory process on the risk of coronary heart disease (CHD) [1]. In this commentary, I discuss recent results in the coronary disease genetics literature pertinent to the findings of Brown *et al*., examine the difficulties inherent in assigning causal roles to the measurements of particular plasma biomarkers of inflammation in CHD and discuss the approach sometimes termed 'Mendelian randomization', which has in recent years been increasingly used to address this issue.

Numerous epidemiological studies and large meta-analyses have convincingly shown evidence for the association between higher plasma levels of a number of the inflammatory proteins coded for by inflammatory genes (most extensively, C-reactive protein [CRP] but also some members of the interleukin family) and CHD risk. However, the associations are less strong than those observed for 'classical' risk factors such as low-density lipoprotein-cholesterol, and the magnitude of the associations observed has tended to be quite sensitive to the degree of adjustment for potential confounding factors that has been made [2-4]. This raises the possibility that the association between such inflammatory proteins and CHD risk is largely, or possibly entirely, due to confounding or 'reverse causality' [5]. Reverse causal bias can arise in situations when subclinical manifestations of a disease process which starts early in life (such as CHD) are already sufficient to cause elevations in candidate biomarkers at the time that a prospective cohort is ascertained, such that an association is observed between baseline levels of the biomarker and subsequent clinical manifestations of the disease process. With respect to CHD and biomarkers of inflammation, higher levels of CRP have been shown even among children who have higher levels of 'classical' risk factors for CHD, such as obesity [6-8]. Of course, if the addition of plasma levels of particular factors to existing risk algorithms significantly improves their predictive utility, this would constitute a clinical advance, even if the association could not be proved to be causal. However, a strong presumption of causality is necessary before a particular gene product could be pursued as a potential therapeutic target. For a number of plausible candidate inflammatory genes, this remains an open question. Establishing robust associations between single nucleotide polymorphisms (SNPs) in such genes which affect the plasma levels of the gene product and CHD would constitute strong evidence in favour of a causal involvement in disease, an approach sometimes termed 'Mendelian randomization' [9].

Genomewide association studies (GWAS) of CHD, in which the genotypes at hundreds of thousands of common genetic variants (SNPs) throughout the human genome are compared in thousands of CHD cases and a similar number of controls have, in recent years, had a substantial degree of success in identifying loci robustly associated with CHD [10-14]. Thus far, around a dozen loci have been detected. Many have associations with plasma lipoprotein levels but some, including the effect that has consistently been identified as the strongest (on chromosome 9p21), are uncorrelated with any of the 'classical' risk factors for CHD. It is perhaps noteworthy that no GWAS has convincingly implicated any of the inflammatory genes that are the subject of Brown *et al*.'s analyses. However, one limitation of the GWAS approach is that the heavy penalty for multiple testing that must be imposed for assessing genotypes at very many loci to some extent limits the power of the approach to detect weak effects. Meta-analysis of GWAS datasets has been carried out in order to attempt to address this issue [15-17]. In the meta-analyses conducted so far, which have included up to 25,000 cases, no strong associations with the inflammatory genes studied by Brown *et al*. have been discovered. However, due to the play of chance, it would not be expected that even such studies would detect all the common variants affecting CHD risk. Moreover, GWAS analyses have, thus far, been confined to examining typed variants one-by-one rather than in ordered series of genotypes along chromosomal segments (haplotypes) during the initial screening stage involving all SNPs. Therefore, effects on risk conferred, not by genotype at one SNP but by the interaction of multiple loci on a haplotype, could have been missed. There is precedent for important effects of this type. For example, the epsilon-2/epsilon-3/epsilon-4 isoform polymorphism in the Apolipoprotein E gene is defined by the genotype at two nonsynonymous SNPs at codons 112 and 158 of the protein. The ApoE isoform polymorphism has been shown in large studies to be strongly associated with CHD risk (with a per-allele risk ratio of around 1.3) [18], and yet SNP-by-SNP analysis in the GWAS studies conducted thus far has consistently failed to identify ApoE as a strong risk gene. Thus, even in the GWAS era, there remains a place for more focused examinations of candidate genes in large cohorts, particularly if haplotype-specific effects are investigated.

Brown *et al*. examined the relationship between 51 common genetic polymorphisms in 35 genes involved in inflammation and the risk of CHD, in a large collection of 2699 siblings from 891 families of whom 1154 had CHD. They utilized statistical tests which took account of the 'overmatched' nature of family as opposed to unrelated controls and adjusted, where possible, for the effects of 'classical' CHD risk factors. In unadjusted analyses, they found evidence for an association between CHD and a long-range three-locus haplotype spanning 52 Kb of the IL-1 gene cluster. The haplotype is common (frequency 0.41), so even the relatively small effect on CHD risk that was found (odds ratio [OR] per copy 1.21; 95% CI [confidence interval] 1.04 - 1.40; *P *= 0.006) could translate into a fairly substantial effect at the population level. However, after full adjustment for classical risk factors, the result was no longer significant. This prompted Brown *et al*. to test whether the association between the IL-1 cluster haplotype and CHD could be mediated through a classical risk factor. Intriguingly, a borderline significant association was observed between hypercholesterolemia and the IL-1 risk haplotype (OR 1.15; 95% CI 1.03-1.29; *P *= 0.01). Brown *et al*. acknowledge the potential for the association with hypercholesterolaemia to be confounded due to the strong correlation between MI and treatment with statin drugs, which was one of the criteria they used to define hypercholesterolaemia. Nevertheless, the suggestion that the IL-1 haplotype adversely affects plasma lipids is one that merits further study, possibly in a quantitative fashion in cohorts without the high level of statin exposure present in the study of Brown *et al*. It is perhaps noteworthy that joint analyses of GWAS data from up to 40,000 individuals have, not, thus far, shown any effect of the IL-1 locus on plasma lipids [19-22], although, in general, haplotype analyses, as noted above, have not featured in the screening stage of such investigations. Genetic polymorphisms identified so far that affect plasma lipid concentrations (with the exception of the effect of polymorphism at the apo(a) locus on plasma levels of lipoprotein(a), which is substantial), account for only a small fraction of the calculated heritability of the lipid traits investigated. Indeed, this same pattern has been observed for other quantitative phentotypes, such as height, and all complex diseases so far studied using GWAS. The majority of the effort to find what has been termed the 'missing heritability' of complex traits is presently focused on the investigation of genetic variation not captured in GWAS studies, such as rare variants using ultra-high throughput sequencing and complex copy number variants. However, the existence of long-range haplotype-specific effects that can be detected by combinations of SNPs, such as that illustrated by Brown and colleagues, is a potential explanation for a proportion of the missing heritability. As the authors state, confirmation of their findings in other cohorts will be of substantial interest in the future.

In conclusion, the findings of this hypothesis-generating study, if replicated, could be of considerable importance, not only in elucidating the relationship between cytokine genes and CHD risk but also, more generally, in signposting the way towards identifying some of the 'missing heritability' that genetic epidemiologists are seeking in the post-GWAS era.

## Abbreviations

CHD: coronary heart disease; CRP: C-reactive protein; GWAS: genomewide association studies; SNP: single nucleotide polymorphism.

## Competing interests

The author declares that he has no competing interests.

## Pre-publication history

The pre-publication history for this paper can be accessed here:

http://www.biomedcentral.com/1741-7015/8/6/prepub
